# Supplementary figures and images for: Exploring Avian Influenza Viruses in Yakutia—The Largest Breeding Habitat of Wild Migratory Birds in Northeastern Siberia
Source: Viruses. 2025 Apr 27;17(5):632. doi: 10.3390/v17050632 (PMC12116004; doi:10.3390/v17050632)

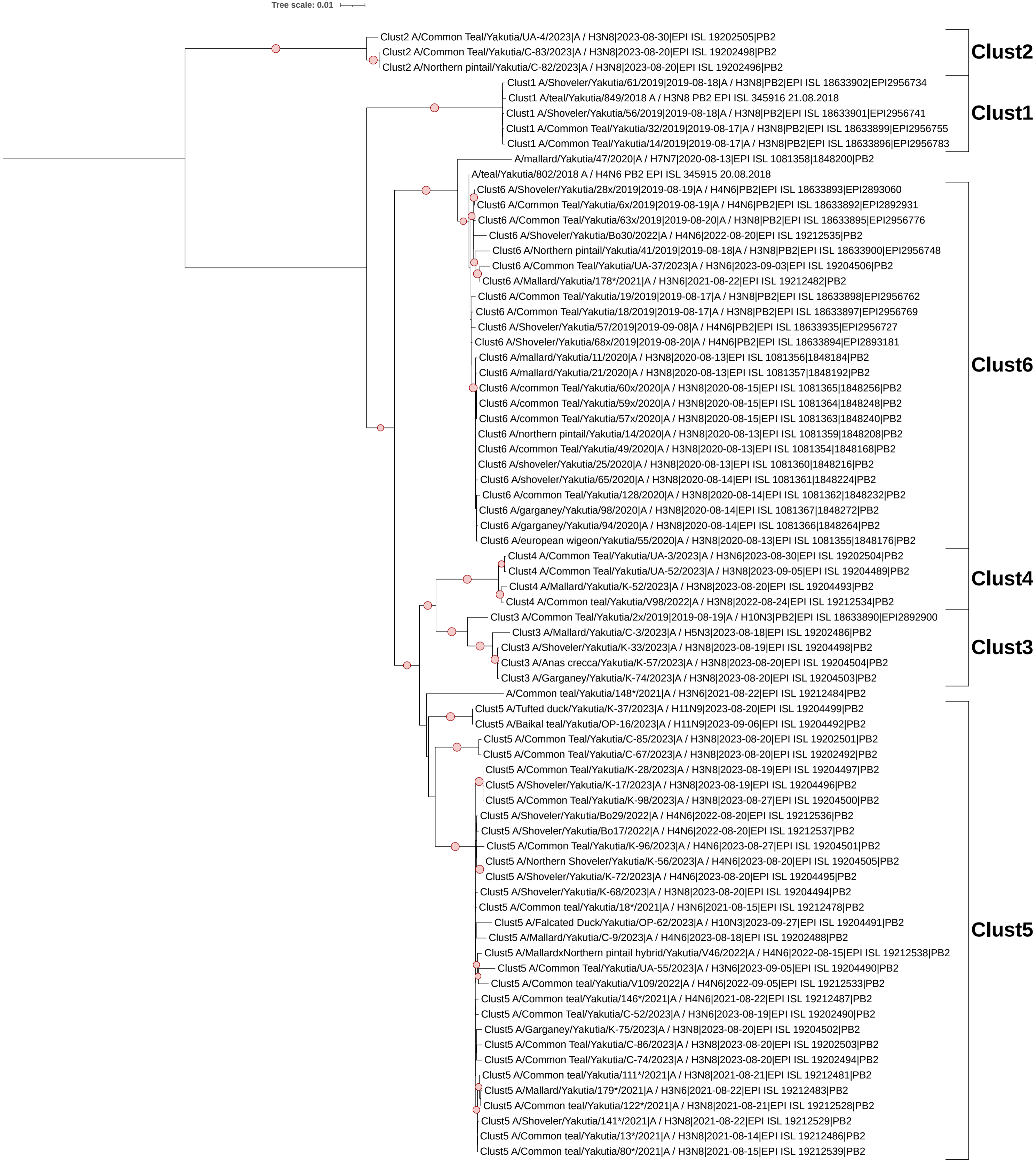

Supplement: Supplementary file 1 [file viruses-17-00632-s001.zip › Figure S1.pdf]

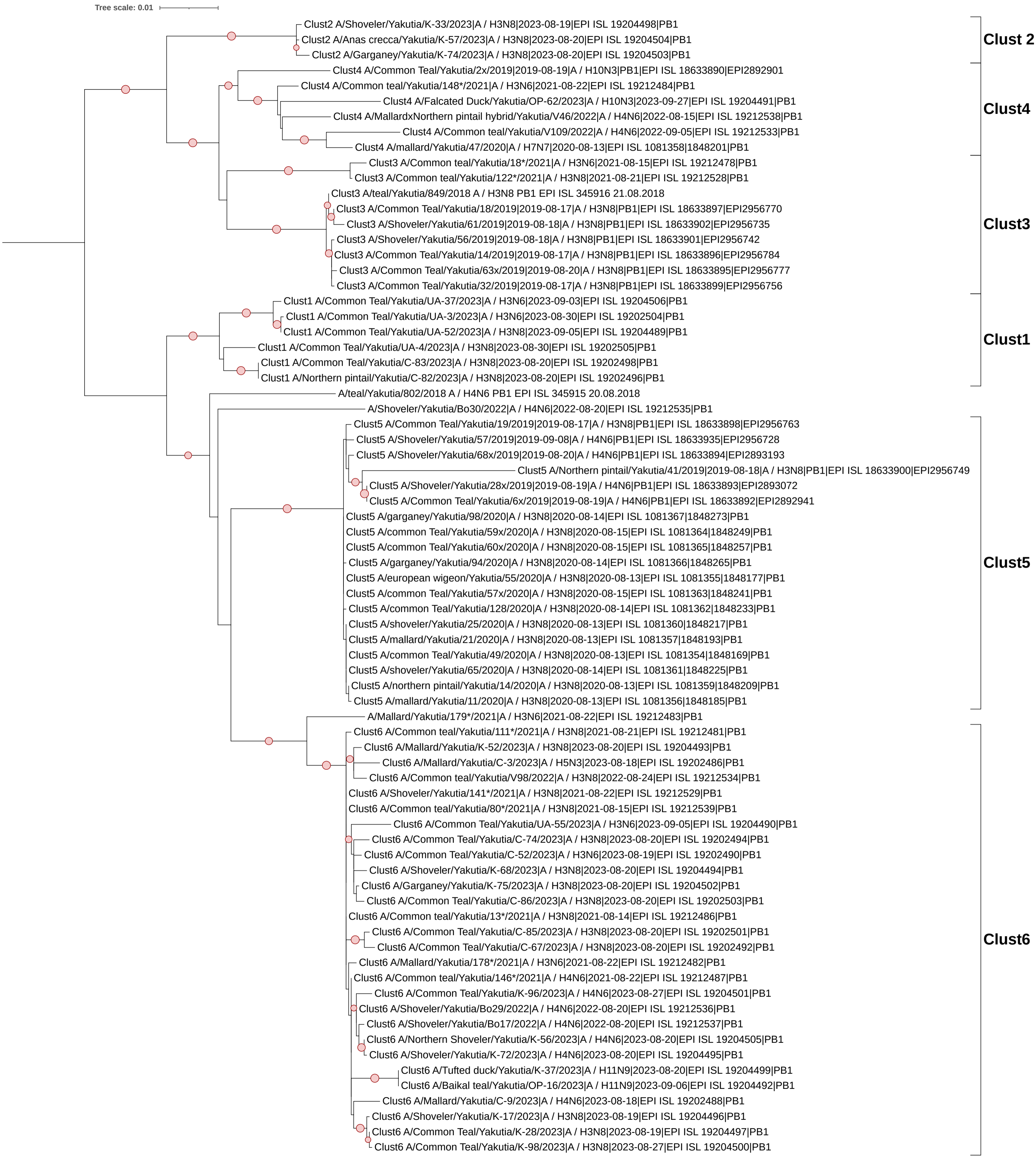

Supplement: Supplementary file 1 [file viruses-17-00632-s001.zip › Figure S2.pdf]

Tree scale: 0.01

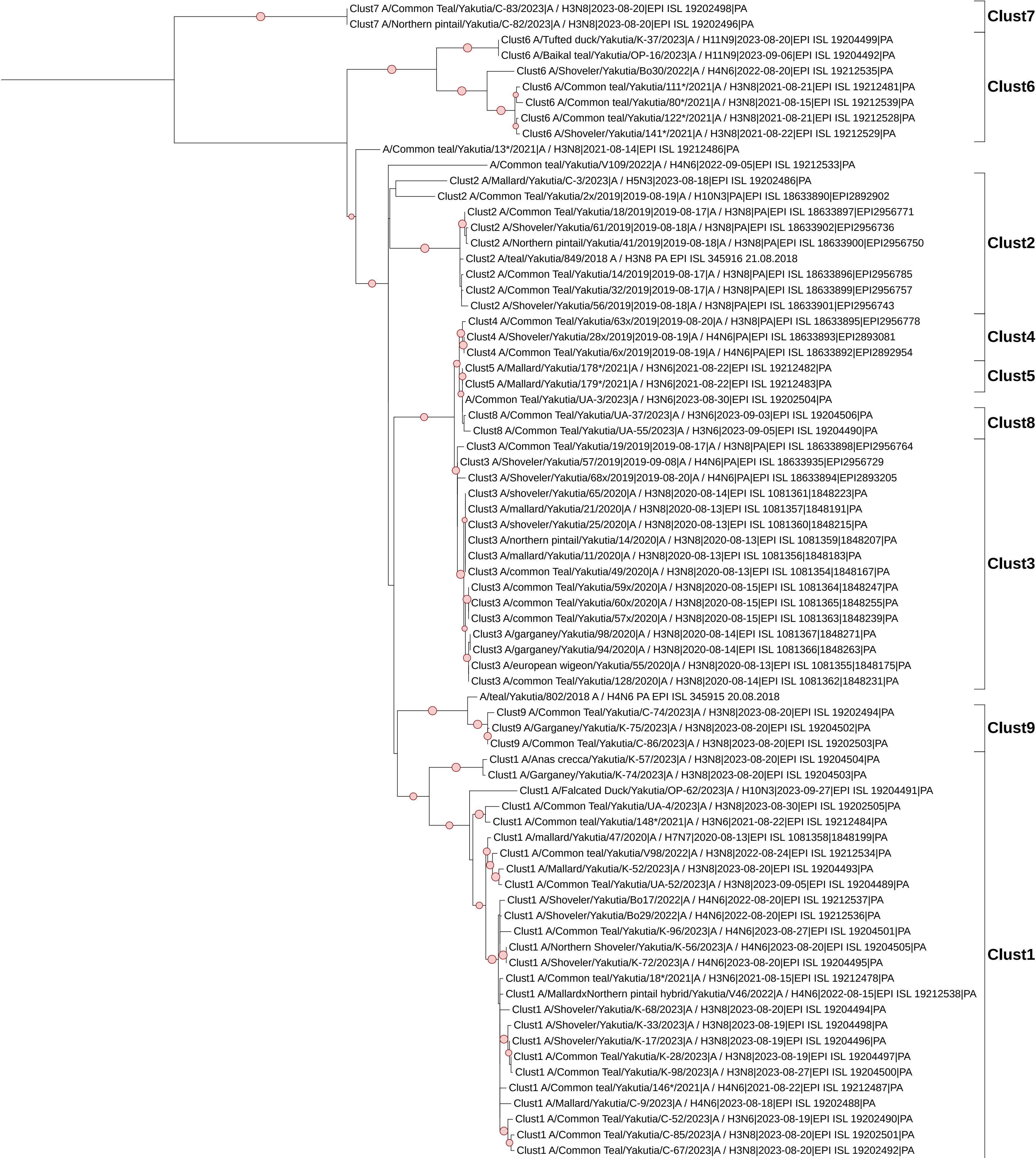

Supplement: Supplementary file 1 [file viruses-17-00632-s001.zip › Figure S3.pdf]

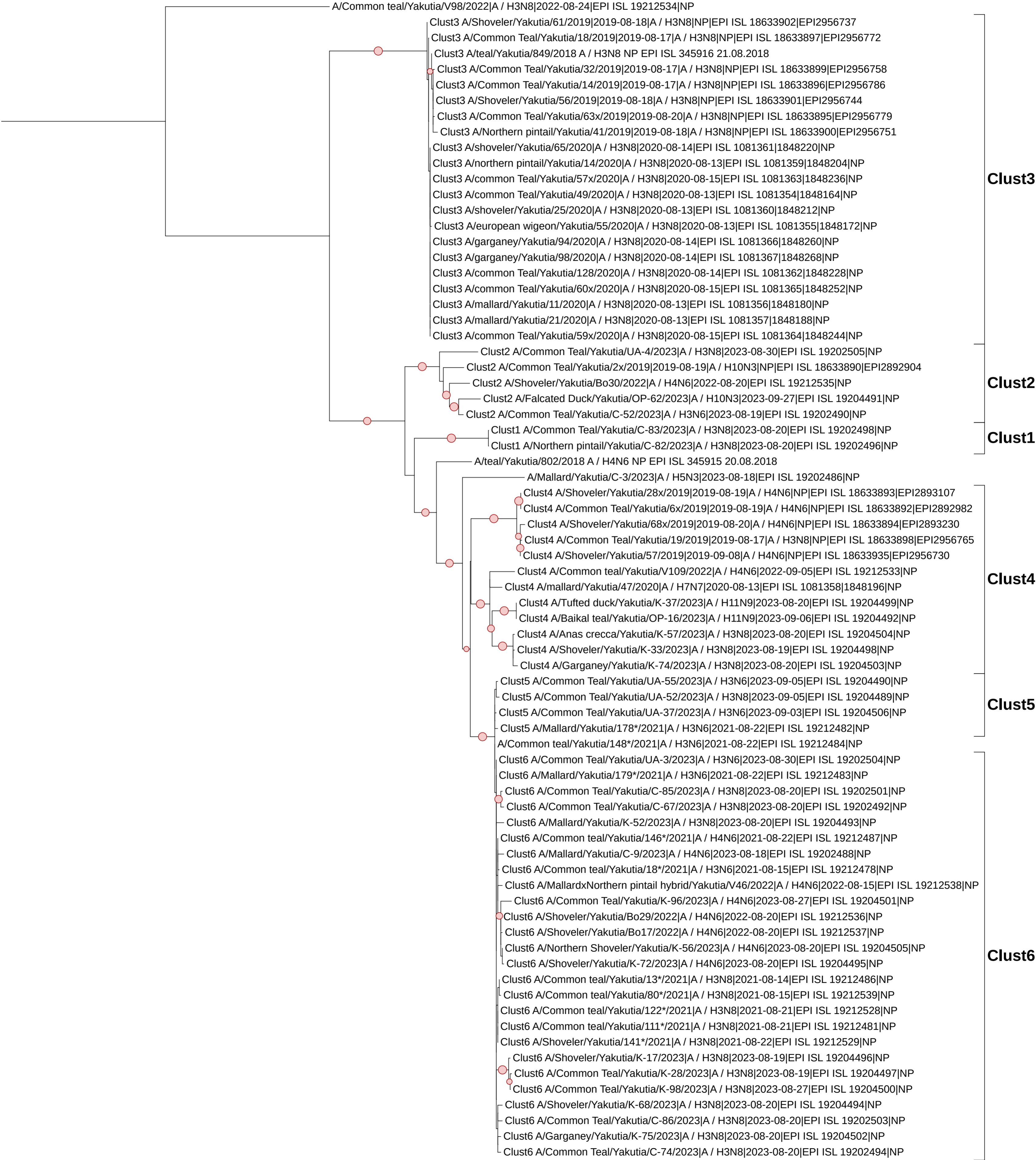

Supplement: Supplementary file 1 [file viruses-17-00632-s001.zip › Figure S4.pdf]

Tree scale: 0.01

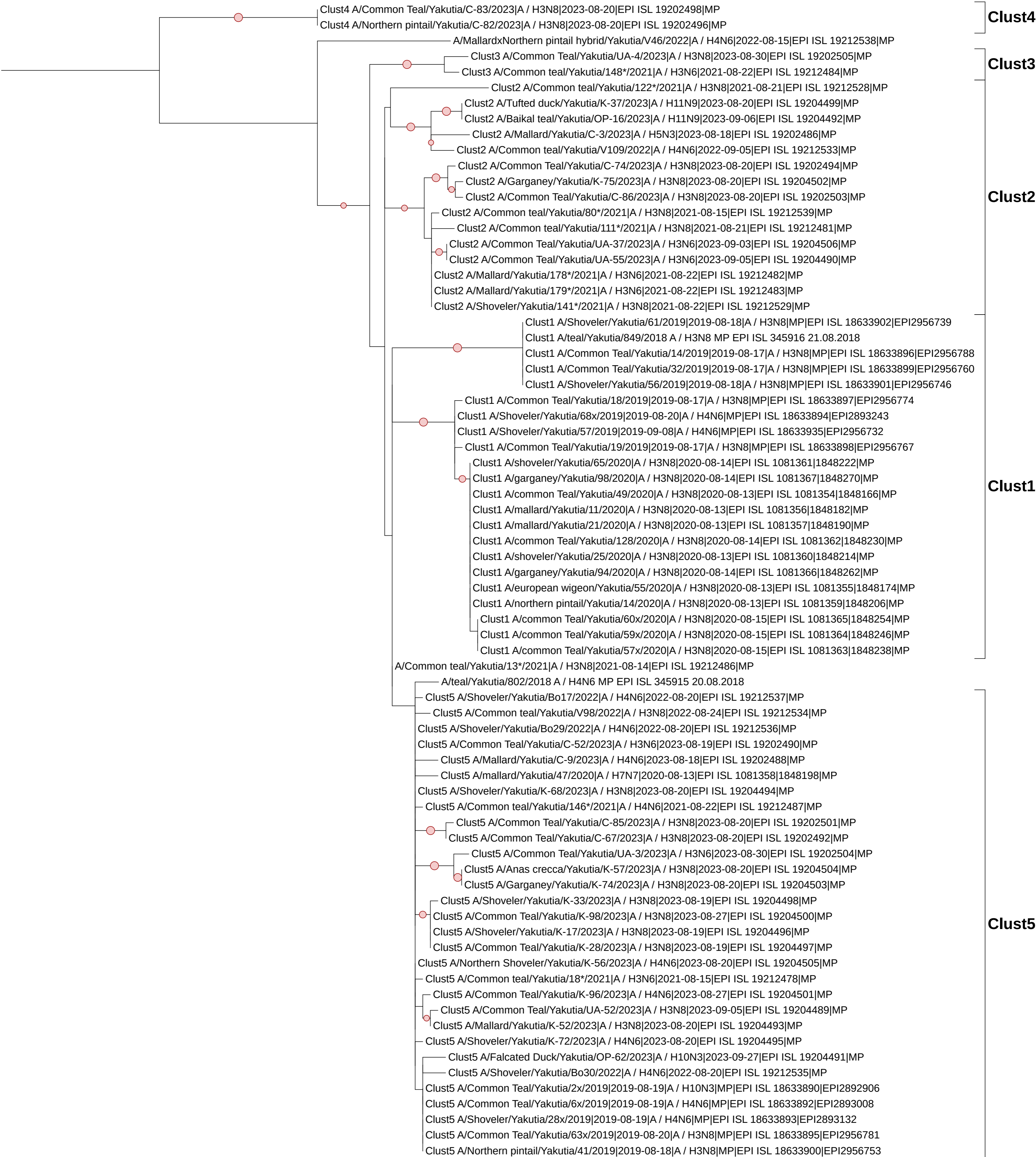

Supplement: Supplementary file 1 [file viruses-17-00632-s001.zip › Figure S5.pdf]

Tree scale: 0.1

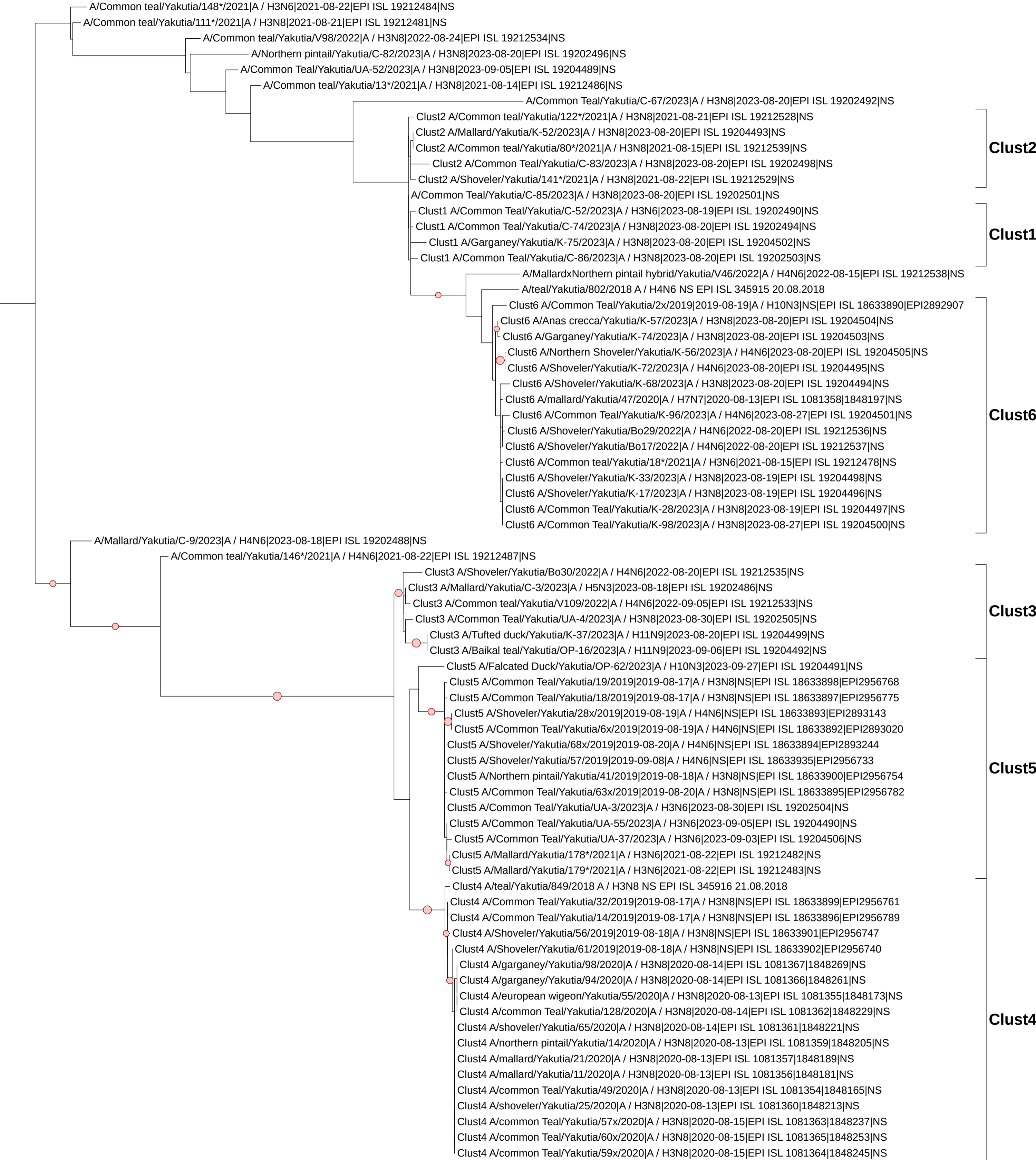

Supplement: Supplementary file 1 [file viruses-17-00632-s001.zip › Figure S6.pdf]
